# Educating the next generation of psychiatrists in the use of clinical neuromodulation therapies: what should all psychiatry residents know?

**DOI:** 10.3389/fpsyt.2024.1397102

**Published:** 2024-05-15

**Authors:** Sahit N. Menon, Tyler Torrico, Bruce Luber, Brian Gindoff, Lisa Cullins, William Regenold, Sarah H. Lisanby

**Affiliations:** ^1^ Noninvasive Neuromodulation Unit, Experimental Therapeutics and Pathophysiology Branch, National Institute of Mental Health, Bethesda, MD, United States; ^2^ Emotion and Development Branch, National Institute of Mental Health, Bethesda, MD, United States

**Keywords:** psychiatry residency, neuromodulation, TMS, ECT, DBS, VNS

## Abstract

A variety of neuromodulation treatments are available today and more are on the way, but are tomorrow’s psychiatrists prepared to incorporate these tools into their patients’ care plans? This article addresses the need for training in clinical neuromodulation for general psychiatry trainees. To ensure patient access to neuromodulation treatments, we believe that general psychiatrists should receive adequate education in a spectrum of neuromodulation modalities to identify potential candidates and integrate neuromodulation into their multidisciplinary care plans. We propose curricular development across the four FDA-cleared modalities currently available in psychiatric practice: electroconvulsive therapy (ECT), transcranial magnetic stimulation (TMS), deep brain stimulation (DBS), and vagus nerve stimulation (VNS). With a focus on psychiatry residency training, the article delineates core learning components for each neuromodulation technique. For each modality, we review the clinical training status, the respective FDA-cleared indications, mechanisms of action, clinical indications and contraindications, adverse effects, informed consent process, dosing considerations, and clinical management guidelines. The approach outlined in this article aims to contribute to the development of a well-rounded generation of psychiatry trainees with the capacity to navigate the growing field of neuromodulation. Whether or not a psychiatrist specializes in delivering neuromodulation therapies themselves, it is incumbent on all psychiatrists to be able to identify patients who should be referred to neuromodulation therapies, and to provide comprehensive patient care before, during and after clinical neuromodulation interventions to optimize outcomes and prevent relapse.

## 1 Introduction

Neuromodulation interventions have long been a core part of psychiatric practice. For example, electroconvulsive therapy (ECT) has been in clinical use since the early 20^th^ Century. Today, these interventions have expanded to include transcranial magnetic stimulation (TMS), deep brain stimulation (DBS), and vagus nerve stimulation (VNS), each of which is cleared by the Food and Drug Administration (FDA) for the treatment of psychiatric disorders. As device-based therapies expand, future psychiatrists must be equipped to integrate these interventions into treatment plans.

Currently, the Accreditation Council for Graduate Medical Education (ACGME) guidelines on neuromodulation competencies in psychiatry residency consist of a single sentence: “indications for and uses of electroconvulsive and neuromodulation therapies (1).” This leaves training programs limited guidance on how to structure neuromodulation training in psychiatry residency.

While other articles discuss the potential role for a subspecialty in interventional psychiatry (2, 3), we focus on what every psychiatrist should know about neuromodulation therapies. We propose that knowledge in neuromodulation techniques should be integrated into general psychiatry residency. Having foundational knowledge of neuromodulation techniques in residency will equip psychiatry trainees with procedural knowledge, empower them to refer patients for neuromodulation when appropriate, and help them understand how to manage patients before, during and after neuromodulation interventions to sustain remission and to prevent relapse.

The 2010 American Psychiatric Association (APA) practice guidelines for treatment of major depressive disorder (MDD) in adults includes ECT and TMS as acute phase treatment modalities. VNS is cited as an option after inadequate response to at least four adequate trials of antidepressants, including ECT (4). In 2016, the Canadian Network for Mood and Anxiety Treatments (CANMAT), published clinical guidelines for management of adults with MDD, which includes ECT, TMS, DBS, VNS, magnetic seizure therapy (MST) and transcranial direct current stimulation (tDCS) (5). Aligning clinical practice with these guidelines requires that psychiatry residents receive neuromodulation training.

Research indicates that one potential barrier to neuromodulation access is inadequate medical education (6). Access to neuromodulation is limited by professionals trained to refer for, prescribe, and administer these modalities. Research has shown that in the inpatient setting, ECT recipients were more likely to be female and white-non-Hispanic (7, 8). Furthermore, Noorani et al. showed that 29% of ECT providers in the US are female, and only 1% are Black or African American (9). Clinician biases may be a potential source of ECT disparities among patients of color (10). In healthcare, medical bias, or the tendency for physicians to make decisions on the basis of prejudice or stereotypes, can lead to lower quality of care (11). To address this issue, training programs can integrate culturally informed treatment strategies that acknowledge how certain practices lead to poor mental health outcomes among minoritized ethnic groups (12). Additional work has shown that ECT is not provided as an inpatient procedure in 9 out of 10 hospitals (13), which may relate to unfamiliarity with the technique and negative attitudes towards ECT. This highlights the importance of early ECT training in psychiatry residency. Other barriers to initiating ECT include lack of well-trained colleagues and ECT practitioners (14). Prior studies have suggested that observing ECT generally reduces negative attitudes (15), which could translate to improved patient access.

Additionally, a survey-based study on psychiatrists’ attitudes towards TMS found that psychiatrists who lacked formal TMS training did not know the indications for referring a patient with depression, and only 1 in 4 knew how to properly refer (16). Patient safety is linked to provider training, as the risk-to-benefit ratio of TMS varies for each individual (17). These findings highlight the importance of TMS education in relation to patient care.

Established guidelines on neuromodulation training, include those outlined by the APA’s 2001 Task Force Report, *The Practice of Electroconvulsive Therapy: Recommendations for Treatment, Training, and Privileging*. The report proposes that psychiatry residents should observe at least ten ECT treatments for three different patients and complete four hours of formal didactic education, which covers clinical indications, administration techniques, and management of adverse effects, among other principles (18). Additionally, an International Federation of Clinical Neurophysiology (IFCN) committee has proposed TMS training guidelines, which include core knowledge of TMS theory and mechanisms, basic skills for TMS use, and safety and ethical concerns (19). Additional literature from the IFCN committee has delineated competencies for clinicians who have completed residency training (20). There are also publications on core competencies for an interventional psychiatry fellowship in noninvasive and invasive neuromodulation – including indications, evaluation, and periprocedural care (21). However, there is limited literature, if any, that proposes competencies across neuromodulation modalities during general psychiatry residency.

Training in brain stimulation aligns with the emerging trend of competency-based medical education (CBME) (22). One avenue for implementation could be through entrustable professional activities (EPAs) (23), which are complementary approaches to CBME that involve targeted assessments. High-fidelity simulation may reduce negative attitudes related to ECT (24) and improve skills and knowledge (25). Psychiatry residents participating in a blended ECT curriculum for late-life depression, including clinical cases, didactic seminars, and hands-on management, expressed high satisfaction (26). Institutions have explored the value of disseminating self-learning modules (SLM) on TMS for treatment-resistant depression, and found that SLMs could serve as effective primers for trainees prior to hands-on training (27). Finally, the modified Delphi process could be utilized to develop consensus in postgraduate ECT and TMS competencies (28, 29).

Ultimately, the approach outlined in this article aims to contribute to the development of a well-rounded and competent generation of future psychiatrists equipped to navigate the complexities of modern neuromodulation techniques in psychiatry.

## 2 Electroconvulsive therapy

### 2.1 Status in clinical psychiatry training programs

Estimates of ECT exposure in psychiatry residency vary, but studies have shown that 75% of residency programs require ECT exposure, though 37% stated that a resident would participate in less than ten treatments (30). Prior work has suggested that insufficient training may prevent psychiatrists from practicing effective medical care, which can reduce patient access to life-altering treatments, including ECT (31).

### 2.2 Current FDA status

ECT is FDA-cleared for severe major unipolar or bipolar depressive episodes and catatonia, for those that are 13 years or older, and whose disorder is “treatment resistant, or requiring rapid response due to the severity of their psychiatric or medical condition (32).” In 2018, the FDA reclassified ECT devices from Class III (high risk) to Class II (moderate risk) (32). Estimates of ECT use vary, but one study evaluating insurance claims found that the ECT utilization rate was 5.56 per 100,000 patients in the general population (33).

### 2.3 Recommended core knowledge

#### 2.3.1 Mechanisms of action

Residents should know that ECT induces controlled seizures by delivering electrical currents to the brain, leading to alterations in neurotransmitter release and synaptic plasticity (34).

#### 2.3.2 Clinical indications and contraindications

ECT is FDA-cleared for severe major depression in unipolar or bipolar disorder (35), and for catatonia. Off-label indications include acute schizophrenia episodes, bipolar manic and mixed episodes. There are no absolute contraindications to ECT, but relative contraindications include unstable medical conditions, active chest infection, recent myocardial infarction, and high intracranial pressure (36).

#### 2.3.3 Adverse effects

Trainees should know ECT’s common adverse effects, including cognitive impairment, headaches, cardiovascular complications, increased intracranial pressure, and muscle soreness (37). However, advancements in ECT technique, such as ultra-brief pulse stimuli, have reduced cognitive side effects.

#### 2.3.4 Informed consent

Psychiatrists should understand how to provide patients with comprehensive information about the procedure, its potential benefits, and risks. Psychiatrists in the United States should know that guidelines regarding voluntary and involuntary ECT vary depending on the state of practice. Informed consent for ECT is preferable. However, patients with catatonia or severe behavioral disorders are often unable to provide informed consent, and these cases should be considered in accordance with state-specific guidelines for involuntary ECT.

#### 2.3.5 Dosing considerations

Dosing parameters for ECT, including stimulus intensity, frequency, pulse width and train duration, are individualized. General psychiatrists should know that ECT dosage is set relative to the threshold for inducing seizure, and titrating seizure threshold allows for precise dosing, minimizing adverse effects while optimizing therapeutic outcomes. An ECT curriculum should also cover the different types of ECT based on electrode placement (i.e., bifrontal, unilateral, bitemporal), pulse width and dosage relative to seizure threshold. A typical acute treatment course is 2 or 3 treatment sessions per week, over 4 weeks. Continuation ECT can occur with a taper to weekly or monthly sessions over six months, after which maintenance ECT is typically provided every 1–12 weeks (38).

#### 2.3.6 Clinical management

Close collaboration between the referring psychiatrist and the ECT team is essential for proper psychopharmacological management before, during and after ECT. Continuation and maintenance ECT, along with combination pharmacotherapy, may help maintain remission after the acute course of ECT. For involuntary ECT, regular communication with the patient’s family or surrogate decision-maker is essential.

## 3 Transcranial magnetic stimulation

### 3.1 Status in clinical psychiatry training programs

The presence of TMS education in psychiatry residency varies, and there is limited research that indicates whether TMS is included in the core educational curriculum.

### 3.2 Current FDA status

TMS gained its first FDA-clearance for depression in 2008 (39). TMS is currently FDA-cleared for the treatment of major depressive disorder, obsessive-compulsive disorder, migraines, smoking cessation, and anxious depression, all in adults (40). Recently, a group of TMS experts in Europe have established clinical recommendations for repetitive TMS (rTMS) protocols (41), and a group in Portugal provided an overview of the regulatory landscape for TMS (42), both of which could inform residency curriculum.

### 3.3 Recommended core knowledge

#### 3.3.1 Mechanisms of action

TMS exerts its therapeutic effects by generating magnetic fields that induce electrical currents in specific brain regions. This stimulation modulates neuronal activity, leading to changes in neurotransmitter release and synaptic plasticity (43).

#### 3.3.2 Clinical indications and contraindications

TMS is commonly used for treatment-resistant depression, after the lack of response to at least one antidepressant medication. Its applications also include the FDA-cleared conditions listed above. Relative contraindications may include the presence of metal implants in the head, a history of seizures, or head trauma (19).

#### 3.3.3 Adverse effects

TMS is generally well-tolerated, with few systemic side effects. Common adverse effects include mild scalp discomfort or headache during or after the procedure. Seizures are a rare adverse event (44).

#### 3.3.4 Informed consent

Informed consent for TMS involves providing patients with information about the procedure, its potential benefits, and possible side effects. Patients should understand the non-invasiveness of TMS, the duration and frequency of sessions, the low risk of systemic side effects and the expected outcomes.

#### 3.3.5 Dosing considerations

TMS dosing involves specifying the stimulation parameters, including identifying a patient’s motor threshold, defining the form of TMS (i.e. monofrequency versus intermittent theta burst (iTBS), its stimulation schedule (i.e. daily versus accelerated protocols), and specifying the type of TMS coil (H-coil versus the figure-8 coil). The general psychiatrist should know that TMS pulse intensity is set relative to the threshold to induce a motor twitch. Failing to titrate motor threshold could lead to a seizure (for excessively high doses) or inadequate treatment (for inappropriately low doses). FDA-cleared protocols specify dosing, coil type, and stimulation schedule. Most TMS treatment schedules are daily sessions over 4–6 weeks (45), but accelerated protocols involve up to ten sessions daily over 5 days (46).

#### 3.3.6 Clinical management

Collaboration between the referring psychiatrist and the TMS care team is vital for effective clinical management. Monitoring treatment response, adjusting stimulation parameters as needed, and addressing any side effects contribute to the success of TMS therapy. Psychiatrists may also consider pairing TMS with psychotherapy, which may benefit certain patients (47). Relapse prevention strategies, such as continued TMS and combination pharmacotherapy, can sustain remission.

## 4 Deep brain stimulation

### 4.1 Status in clinical psychiatry training programs

Exposure to DBS in psychiatry residency training has not been quantified in the literature to our knowledge. However, one may expect that exposure to DBS is less than that to ECT and TMS due to the invasive nature of the treatment. DBS is reserved for cases with significant treatment resistance, which may also contribute to a limited knowledge base among psychiatrists. DBS management requires specialized interventional units to facilitate collaboration between neurosurgeons, psychiatrists, and nursing staff.

One survey of psychiatrists and psychiatry residents in North America suggested practitioners were more likely to refer patients for DBS as a therapeutic option over other forms of functional neurosurgery; however, the number of referrals remains very low due to the group citing a lack of procedural knowledge and safety concerns (48).

### 4.2 FDA clearance status

At the time of this writing, in psychiatry, DBS has only received FDA clearance for treatment-resistant obsessive-compulsive disorder under the Humanitarian Device Exemption (HDE H050003) in 2009 (49).

### 4.3 Recommended core knowledge

#### 4.3.1 Mechanisms of action

DBS involves neurosurgical implantation of electrodes that deliver electricity to targeted regions of the brain to modulate neural circuits. DBS induces changes in neurotransmitter systems and can modulate neural pathways associated with psychiatric symptoms. DBS may suppress pathological neural circuits or facilitate the development of new synaptic connections (50).

#### 4.3.2 Clinical indications and contraindications

Treatment resistant obsessive-compulsive disorder is currently the only FDA-cleared psychiatric treatment of DBS. Contraindications include medical comorbidities that would make a patient a poor surgical candidate, in addition to substance use, personality disorders, cognitive impairment, and brain pathology that would interfere with the effectiveness of DBS (i.e tumor, stroke, cerebral atrophy) (51).

#### 4.3.3 Adverse effects

Similar to other neurosurgical interventions, potential adverse effects of DBS include surgical complications, headaches, fatigue, and mood fluctuations (52).

#### 4.3.4 Informed consent

Obtaining informed consent for DBS is a nuanced process, which should involve consultation with DBS experts and the neurosurgeon in an interdisciplinary manner.

#### 4.3.5 Dosing considerations

Psychiatry residents should be aware that individualized dosing is a critical aspect of DBS therapy. Adjustments can be made to the frequency, intensity, pulse width and location of stimulation. Titration of dose is usually made over 3 to 6 months based on the clinical response (53). DBS dosing should be managed by DBS experts and general psychiatrists should plan to have regular communication with the DBS team to optimize clinical outcomes.

#### 4.3.6 Clinical management

DBS candidates already have a high degree of symptom severity and chronicity. Optimal collaboration requires that psychiatrists recognize both symptom improvement and adverse effects, and that neurosurgeons are equipped to make relevant surgical decisions. Due to the invasiveness and interdisciplinary nature of the procedure, trainees should know when to appropriately refer for DBS. Long-term care requires vigilance for signs of relapse (that might signal the need for parameter adjustment or battery replacement) and optimizing pharmacological and behavioral interventions to maximize functioning.

## 5 Vagus nerve stimulation

### 5.1 Status in clinical psychiatry training programs

To the best of our knowledge, there is limited emphasis on VNS training, if any, in psychiatry residency training programs. Furthermore, literature suggests that psychiatrists lack adequate exposure to VNS as a form of therapy, possibly due to its limited use (54).

### 5.2 FDA clearance status

VNS was FDA-cleared for the treatment of epilepsy in 1997 (55), and for treatment-resistant depression in 2005 (56). VNS is on-label for adults with chronic or recurrent illness (defined as ≥2 years) who are treatment resistant (defined as not responding to ≥4 adequate medication trials) as an adjunctive treatment (e.g. add-on to medications).

### 5.3 Recommended core knowledge

#### 5.3.1 Mechanisms of action

VNS involves surgical implantation of the VNS device on the chest under the left collarbone and surgical access in the neck to the left vagus nerve where electrodes connect to the generator in the chest. The electrodes deliver stimulation to the vagus nerve, which modulates neurotransmitters and induces neuroplastic changes (57).

#### 5.3.2 Clinical indications and contraindications

VNS is FDA-cleared for the adjunctive long-term treatment of chronic treatment resistant unipolar and bipolar depression. Relative contraindications include poor surgical candidates, prior resection of the vagus nerve, personality disorders, substance use, severe arrythmias, history of vasovagal syncope, history of asthma or obstructive sleep apnea, hoarseness, or gastric or duodenal ulcer (58).

#### 5.3.3 Adverse effects

During stimulation, adverse effects include hoarseness and voice alteration. Dyspnea, cough and neck discomfort are also possible side effects. Transient laryngo-tracheal dysfunction, associated with stimulation of the recurrent laryngeal nerve, occurs transiently in 66% of cases (59). True vocal cord paralysis occurs transiently in 1–2.7% of cases (59). Implantation of the device has a potential for surgical complications, including infection and hemorrhage (60).

#### 5.3.4 Informed consent

Psychiatrists should know that informed consent for VNS is a multidisciplinary process involving VNS experts and neurosurgeons. Patients should understand the implications of having an implanted device, which includes regular monitoring and modifications to device parameters.

#### 5.3.5 Dosing considerations

VNS stimulation parameters include pulse width, current intensity, frequency, on-off time, and duration. Parameters are individualized based on the clinical response (61). General psychiatrists should work closely with VNS experts in each case.

#### 5.3.6 Clinical management

VNS is traditionally considered a long-term approach to treating depression (62), so psychiatrists should evaluate whether they can ensure continuity in potential VNS candidates. Similarly to DBS, regular monitoring of stimulation parameters and addressing adverse effects is important. It is also important to remember to turn the VNS off should the patient need ECT, because it is an anticonvulsant.

## 6 Next generation neuromodulation

This manuscript focuses on currently FDA-cleared neuromodulation technologies, but this is a rapidly evolving field. Experimental noninvasive brain stimulation (NIBS) therapies include MST, transcranial electrical stimulation (tES) [e.g., tDCS, transcranial alternating electrical current stimulation (tACS)], transcutaneous VNS (tVNS), cranial electrotherapy stimulation (CES), low intensity focused ultrasound (LIFU), temporal interference stimulation, transcranial photobiomodulation, and pulsed electromagnetic field therapy. At this time, these techniques require further investigation for use in psychiatry.

Adequate training of psychiatry residents in neuromodulation prepares them to review literature to stay abreast of new developments and make evidence-based recommendations to their patients. This includes future FDA-cleared devices and the latest guidance on expanded clinical indications and optimization of dosing parameters for currently approved devices.

## 7 Conclusion

In summary, as the field of neuromodulation develops, training psychiatry residents in the appropriate use of techniques like ECT, TMS, DBS, and VNS should be an essential part of residency curriculum. Improving neuromodulation education may increase patient access to these therapeutic approaches in the future. Whether or not a psychiatrist chooses to specialize in delivering neuromodulation therapies, it is incumbent on all psychiatrists to identify patients who should be referred to neuromodulation therapies, and to provide comprehensive patient care during and after clinical neuromodulation interventions to optimize outcomes and prevent relapse.

## Data availability statement

The original contributions presented in the study are included in the article/supplementary material. Further inquiries can be directed to the corresponding author.

## Author contributions

SM: Conceptualization, Writing – original draft, Writing – review & editing. TT: Conceptualization, Supervision, Writing – review & editing. BL: Supervision, Writing – review & editing. BG: Writing – review & editing. LC: Supervision, Writing – review & editing. WR: Conceptualization, Supervision, Writing – review & editing. SL: Conceptualization, Supervision, Writing – review & editing.
